# A Systematic Review of Effects of Concurrent Strength and Endurance Training on the Health-Related Quality of Life and Cardiopulmonary Status in Patients with HIV/AIDS

**DOI:** 10.1155/2013/319524

**Published:** 2013-04-03

**Authors:** Mansueto Gomes Neto, Cecília Ogalha, Antônio Marcos Andrade, Carlos Brites

**Affiliations:** ^1^Departamento de Biofunção, Curso de Fisioterapia, Universidade Federal da Bahia (UFBA), 40110-160 Salvador, BA, Brazil; ^2^Programa de Pós-Graduação em Medicina e Saúde da Universidade Federal da Bahia (UFBA), 40110-160 Salvador, BA, Brazil

## Abstract

*Purpose*. To determine the effects of concurrent strength and endurance training (concurrent training) on the Health-Related Quality of Life (HRQOL) and cardiopulmonary status among HIV-infected patients, using a systematic search strategy of randomized, controlled trials (RCTs). *Methods*. A systematic review was performed by two independent reviewers using Cochrane Collaboration protocol. The sources used in this review were Cochrane Library, EMBASE, LILACS, MEDLINE, PEDro and Web of Science from 1950 to August 2012. The PEDro score was used to evaluate methodological quality. *Result*. Individual studies suggested that concurrent training contributed to improved HRQOL and cardiovascular status. Concurrent training appears to be safe and may be beneficial for medically stable adults living with HIV. The rates of nonadherence were of 16%. *Conclusion*. Concurrent training improves the HRQOL and cardiopulmonary status. It may be an important intervention in the care and treatment of adults living with HIV. Further research is needed to determine the minimal and optimal duration, frequency, and intensity of exercise needed to produce beneficial changes in the HIV-infected population subgroups.

## 1. Introduction 

The introduction of highly active antiretroviral therapy (HAART) has dramatically reduced mortality and morbidity in HIV-infected patients. On the other hand HIV-infected patients are experiencing an increasing frequency of noninfectious problems, which can significantly impair the benefits of HAART [1, 2]. 

Exercise training improves and maintains health and reduces the risk of chronic disease in healthy adults [3]. Exercise has been considered an important adjuvant therapy for health promotion of patients with HIV [4, 5]. The proper exercise prescription must take into consideration the choice of exercise's type, in accordance with the objective to be achieved. This includes other important parameters such as intensity, volume, frequency, and duration of exercise [6].

Resistance training has been employed as a therapeutic tool in patients with HIV and is considered safe and effective in improving muscle strength and body composition [7, 8]. Aerobic exercise promotes a significant effect in improving aerobic capacity, measured by maximal oxygen consumption in this population [9, 10].

Recently, the combination of two exercise modalities: concurrent strength and endurance training (concurrent training) has been employed, as recommended by the American College of Sports Medicine [11]. Participation in concurrent training has been recommended for healthy people and adults with chronic medical conditions [12]. 

The physiological stimuli directed to skeletal muscle as a result of strength training or endurance training are divergent in nature, due to competition in metabolic adaptation to exercise. As a consequence, its effects may be limited when compared to training, in terms of specific parameters [13, 14], but in populations with multiple functional impairments the combination of different modes of exercise is part of rehabilitation programs [15, 16].

Some studies have shown a significant improvement in components of muscle performance and endurance during concurrent training in patients with HIV/AIDS [17–19]. The impact of training on functional capacity and mainly on the HRQOL has not been well documented. In addition, there is no consensus among studies regarding the association of the exercise types, or on what is the best intensity of exercise to be prescribed for this population, with little emphasis on HRQOL. This is an open question and a barrier to a large scale use of such strategies in clinical practice.

The goal of this systematic review was to analyze the impact of concurrent strength and endurance training termed concurrent training on HRQOL and cardiopulmonary status of patients living with HIV/AIDS and discuss their implications for clinical practice.

## 2. Methods 

### 2.1. Data Sources and Searches

We performed a computer-based search querying Ovid MEDLINE (1950 to August 2012), LILACS (up to August 2012), CINAHL (Cumulative Index to Nursing and Allied Health, 1982 to August 2012), EMBASE (1980 to August 2012), PEDro (Physiotherapy Evidence Database), and the Cochrane Central Register of Controlled Trials for original research articles published in English, Spanish, and Portuguese. We also performed a manual tracking of citations in the selected articles.

The design group included the terms randomized controlled trials, clinical trials, and controlled trials. The HIV group included the terms human immunodeficiency virus, acquired immunodeficiency syndrome, HIV, HIV infections, HIV long-term survivors, AIDS, and HIV/AIDS. The exercise group included the terms exercise, training, physical exercise, fitness, strength training, progressive resistive/resistance aerobic, aerobic training, concurrent strength and endurance training, concurrent training, anaerobic, exercise therapy, or physical training.

The outcome measures group included the terms quality of life, health-related quality of life, life expectancy, and cardiopulmonary status.

### 2.2. Study Selection

#### 2.2.1. Types of Studies and Participants

We included randomized controlled trials (RCTs) comparing concurrent training with nonconcurrent training or with another exercise modality, performed at least two times per week and lasting at least four weeks. Studies of adults (18 years and older), regardless sexes, at all stages of infection were included. 

#### 2.2.2. Types of Interventions

The concurrent training was defined as the application of aerobic and resistance exercise in the same training session, performed at least two times per week for at least four weeks. Resistance training was defined as exercise that requires muscle contraction against resistance. Aerobic exercise was defined as a regimen containing aerobic interventions (walking, treadmill, cycling, rowing and stair stepping). Exercise programs were described with respect to type of exercise, volume, intensity, frequency, and duration.

#### 2.2.3. Types of Outcome Measures

Cardiopulmonary measures considered in this review included but were not limited to maximal/peak oxygen consumption (V02 max/peak) (mL/kg/min), oxygen pulse (02pulse), maximum heart rate (HR_max⁡_) (beats/min), fatigue (time on exercise), and dyspnea (rate of perceived exertion).

To assess the quality of life related to health we included in the review studies that reported HRQL through standardized and validated scales or questionnaires. 

#### 2.2.4. Data Extraction and Quality Assessment

One reviewer made the search and the initial selection of potentially relevant studies that met the inclusion criteria and two independent reviewers selected the articles that fulfill the inclusion criteria, using a standard form adapted from the Cochrane Collaboration [20] model for data extraction, considering (1) aspects of the study population, such as average age and gender, (2) aspects of the intervention performed, (sample size, type of exercise performed presence of supervision, frequency, and duration of each session), (3) followup, (4) loss of followup, (5) outcome measures and (6) results presented.

There are several scales for assessing quality of RCTs. The PEDro scale assesses the methodological quality of a study based on other important criteria, such as concealed allocation, intention-to-treat analysis, and adequacy of followup. These characteristics make the PEDro scale a useful tool to assess the methodological quality of physical therapy and rehabilitation trials [21].

The PEDro scale [22] is based on a Delphi list [23] and consists of 11 items. The first item is related to external validity and is generally not used to calculate the method score, leaving a score range of 0 through 10 [22]. Most trials had already been rated at least twice by trained evaluators of PEDro database (http://www.pedro.fhs.usyd.edu.au/). If a trial was not included in PEDro or had not been previously rated twice, it was rated independently by two investigators. Studies were excluded in subsequent analysis if the cutoff of 4 points was not reached.

## 3. Results

We identified a total of 98 articles with the search strategy applied to the databases MEDLINE, Scielo, AMED, Lilacs, and PEDro. These 37 items were sent to reviewers for evaluation, selection, and inclusion in the review. Twenty-six were excluded, and 11 papers met entry criterion according to reviewers. Three additional studies were excluded after retrieving the full text. Of these, 2 were RCTs that did not examine outcomes of interest to this review and one study was a duplicate of Mutimura et al. [24].

The remaining eight articles were fully analyzed and approved by both reviewers and had the extraction of data from each RCT (Mutimura et al., 2008 [24]; Hand et al., 2008 [25]; Pérez-Moreno [26]; Dolan et al., 2006 [27] Fillipas et al., 2006 [28]; Driscoll et al., 2004 [29]; Rojas et al., 2003 [30]. Rigsby et al., 1992 [31]).

Each of the papers was assessed using the PEDro scale methodology by both reviewers, with the pre-defined cutoff [4].

### 3.1. Characteristics of the Sample

The initial sample size for the selected studies ranged from 35 [30] to 100 [24]. The final sample ranged from 31 [31] to 97 [24], and mean age of participants ranged from 18 to 60 years. The studies included patients of both genders, but there was a predominance of males. All studies analyzed in this review included outpatients diagnosed with HIV, and the majority of these were under antiretroviral therapy. 

Participants included adults infected with HIV at various stages of the disease with CD4 counts ranging from <100 to >500 cells/mm^3^. Also included were patients with elements of wasting syndrome (either >5% or >10% involuntary weight loss or body weight <90% ideal body weight).

### 3.2. Outcomes of Included Studies 

#### 3.2.1. Cardiopulmonary Status

Stress test was used with a treadmill, stationary bike, and cycle ergometer. Submaximal tests were also used, as the Shuttle test, Kasch Pulse Recovery Test, and six-minute walk test.

#### 3.2.2. Health-Related Quality of Life

WHOQOL-BREF and MOS-HIV health surveys were the tools used to evaluate HRQOL.  Table 1 presents summary data from the 8 RCTs eligible for this systematic review.

### 3.3. Characteristics of Intervention Programs

The exercise intervention characteristics of included studies are provided in Table 2. The parameters used in the application of aerobic and resistance exercise have been reported in most studies, and all described the progressive nature of the training. 

The duration of intervention programs with concurrent training ranged from 6 [25] to 24 weeks [24], but in most studies reviewed, the application period ranged from 12 to 16 weeks. Regarding the length of the session, there was a variation from 60 [27, 28] to 120 [29] minutes. The frequency of sessions varied from two to three times a week. 

For resistance training only two studies [27, 29] specify the type of muscle contraction performed during training: the exercise was performed with concentric and eccentric contractions lasting 6 to 10 seconds, with use of machines, weight stations, and free weights in six studies, but in two, there was no description of the type of equipment used [24]. The exercise intensity was based on the extent of maximum repetition (MR), ranging from 50 to 80% of MR in most studies. One study did not report the prescribed exercise intensity [24]. The application volume of exercise ranged from 1 to 3 sets of 6–18 repetitions. The volume of exercise was not described in one study [24].

For the application of aerobic exercise, all studies reported the treadmill, bike, walking, or joging. Except for the study of Rigsby et al. [31], all reported the criteria for progression training. In all studies the intensity was adjusted based on heart rate (HR_max⁡_), ranging from 45 to 80% HR_max⁡_. 

### 3.4. Effects of Intervention Programs 

#### 3.4.1. Cardiopulmonary Status

Seven studies reported significant improvement in the concurrent training group compared to control group. One study did not compare the improvement intergroups, because they used a before and after evaluation [30].

In the study of Mutimura et al. [24], Shuttle's test was used to evaluate the functional capacity to predict maximum oxygen uptake (VO_2max⁡_). It was improved from 4.7 ± 3.9 to 0.5 ± 0.3 mL/kg per min in the intervention group compared to control (*P* < 0.001). In the study of Fillipas et al. [28], the Kasch Pulse Recovery test (which evaluates the beats per minute after 3 minutes of stepping) was used to assess the endurance, with a lower HR meaning better conditioning. HR was reduced from 19.6 ± 0.6 to 11.7 ± 2.9 in the exercise group compared to control (*P* < 0.001). In the study of Hand et al. [25], there was an improvement of 21% in VO_2_ estimated in the exercise group while there was no improvement in the control group (*P* < 0.001).

Dolan et al. [27] observed an improvement (1.5 ± 0.8 versus −2.5 ± 1.6 mL/kg min^−1^, *P* < 0.001) in VO_2max⁡_ in the training group compared to control. In a study by Driscoll et al., fitness assessment was performed using the time to perform the exercise on a cycle ergometer, with a significant increase in the exercise group compared to control (3 ± 0  0 ± 4 min versus 1.1 min, *P* < 0.001). Rigsby et al. [31] also used maximum time exercise as a parameter for fitness assessment, and he observed a maximum execution time of 1388.46 ± 224.45 versus 965.91 ± 136.14 s in the exercise group and control group, respectively (*P* < 0.001). 

In the study by Rojas et al. [30], a significant improvement in VO_2max⁡_ after training was observed, compared to baseline.  Table 3 provides details of the effects of intervention programs.

#### 3.4.2. Health-Related Quality of Life 

Four researches included HRQOL outcome between the endpoints. All reported significant improvement in HRQOL of the concurrent training group compared to control group.

Mutimura et al. [24] assessed HRQOL using a short-form instrument (WHOQOL-BREF) of the WHO Quality of Life HIV (WHOQOL-HIV). The psychological (1.3 ± 0.3 versus 0.5 ± 0.1; *P* < 0.0001), independence (0.6 ± 0.1 versus 0.0 ± 0.0; *P* < 0.0001), social relationships (0.6 ± 0.2 versus 0.0 ± 0.0; *P* < 0.0001), HIV HAART-specific (1.4 ± 0.2 versus −0.1 ± 0.2; *P* < 0.0001), and QoL domains (0.5 ± 0.3 versus 0.0 ± 0.3; *P* < 0.05) significantly improved in the concurrent training compared to control group.

In the Pérez-Moreno et al. study [26], although statistical significance was not reached for the combined effect of group and time (*P* = 0.09), QOL significantly increased (*P* < 0.01) in the training group after the intervention period, whereas no change was observed in controls.

In the study of Fillipas et al. [28], and Rojas et al. [30], HRQOL was assessed using the Medical Outcomes Study HIV Health Survey (MOS-HIV). In the first study [28] HRQOL showed a between-group difference in only two out of the eleven dimensions. The experimental group improved their overall health while the control group showed slight reduction in this parameter, resulting in a between-group difference of 20.8 points (95% CI 2.0 to 39.7, *P* = 0.03). The experimental group improved their cognitive function while the control group stayed much the same; the between-group difference was 14 points (95% CI 0.7 to 27.3, *P* = 0.04). 

In second study [30] six domains were assessed (health status, global quality of life, energy, physical strength, social contact, and emotional well-being); concurrent training group showed better results than controls in five domains. The only unchanged domain was social contact (*P* > 0.05).

#### 3.4.3. Adherence to Exercise Program

Adherence to exercise is the ability to maintain a program for a certain time. In all studies a varied proportion of patients are excluded before the end of program. In this review, from 471 patients that entered the protocol, only 396 (84%) remained on study at closure.

Mutimura et al. [24] showed the lower rate of discontinuation, with only 4% of withdraw. Conversely, the study of Hand el al. [25] presented the greatest loss of patients in the exercise group, starting with 44 and ending with 21 patients, with loss of 53.3%. The proportion of loss to exercise and control groups was 19.1% versus 11.44%, respectively. 

## 4. Discussion

This systematic review demonstrated that there is sufficient evidence to support the inclusion of concurrent training for adults living with HIV/AIDS.

It is evident that the effectiveness of concurrent training improves aerobic capacity in this population. Despite major differences in exercise prescription and duration of different programs, the aerobic capacity was significantly improved. These findings coincide with results of previous studies that found significant improvements in cardiopulmonary fitness [32, 33].

The effect of concurrent training on QOL is less clear. Only one study showed significant improvement in all domains, while two studies showed impact in specific domains, and in one study the statistical significance was not reached for the combined effect of group and time. This discrepancy can be due to intensity, frequency, and duration of the programs in the analyzed studies, which can result in a different impact on such parameters.

Physical therapists can play an important role in diagnosis and management of the physical dysfunction in HIV-infected patients [34, 35]. This systematic review suggests that concurrent exercise may be an important intervention in the care and treatment of adults living with HIV. Performing concurrent exercise for at least 60 minutes, three times per week for at least six weeks, may contribute to improvements in selected outcomes of cardiopulmonary status. These physiological adaptations to concurrent strength and endurance training may decrease functional limitations and reduce physical disability resulting from HIV infection and increase of HRQOL [35]. 

Intervention strategies should focus on increasing exercise, considering cessation of smoking, dietary counseling, and treatment of arterial blood hypertension and dyslipidemia [36]. Research supports the use of therapeutic exercise as an adjunct therapy in the treatment of symptoms of HIV infection [37].

The number of weekly exercise sessions should be increased until the patient can tolerate three to five sessions weekly. Aerobic exercise should be performed at a moderate intensity: from 11 to 14 on the Borg Rating of Perceived Exertion Scale, at 50% to 85% of peak heart rate, or at 45% to 85%  VO_2max⁡_. Resistance training should focus on large muscle groups, such as the chest, biceps brachia, quadriceps, and hamstrings. The intensity should be moderate (set at 60% to 80% of the one MR) and progressively increased. Overload should be selected with the level this patient can comfortably perform, 8 to 12 repetitions [17]. 

The role of a well-planned exercise program should therefore be emphasized and used as medical treatment among patients and health care professionals. When implementing therapeutic exercise programs for HIV-infected patients, it is recommended that programs be individualized on the basis of the functional capacity and individual symptoms presented by each patient [37, 38]. 

A patient participating in an exercise intervention should be monitored by a physical therapist qualified health-care provider for potential changes in their health status, especially those in more advanced stages of immunosuppression, to prevent any potential adverse events of exercise [39].

Adherence to exercise is an underresearched area with regards to HIV treatment. Very few studies have been reported on the adherence of HIV patients to exercise in the clinical setting. The strongest motivators of adherence to exercise have been shown to be self-efficacy (the concept that a person is capable of performing a course of action to attain a desired outcome) and outcome expectation (the belief that specific consequences will result from specific personal actions) [40, 41].

Further research into reasons for nonadherence as well as for dropouts would be beneficial. In order to gain the most from the exercise, combined exercise programs including targeted psychological support might be the way forward [42].

Future research needs to identify which patient subgroups might benefit the most, the optimal exercise dose needed to lessen disease-related symptoms and maximize clinical benefit, and the effects with different types of programs.

Meta-analyses were not performed due to variability of characteristics of the studies pertaining to the exercise, variation among individual studies in the types of interventions the differences in endpoints, assessment instruments, and variables of exercise prescription.

## 5. Conclusion

Concurrent training is efficacious in treating disability in outpatient men who are HIV positive and showed to be a safe and beneficial intervention in the treatment. Exercise improves aerobic cardiopulmonary status and HRQOL. It may be an important intervention in the care and treatment of adults with HIV.

## Figures and Tables

**Table 1:** Characteristics of the outcomes and results of concurrent training in the trials included in the review.

Study	Patients	Outcomes	Measures		Results	
			Aerobic capacity	HRQOL	Aerobic capacity	HRQOL
Mutimura et al., 2008 [24]	HIV	Aerobic capacity HRQoL	Shuttle test	WHOQOL-BREF	↑VO_2peak_	↑QOL

Hand et al., 2008 [25]	HIV	Aerobic capacity	Graded exercise stress test	NA	↑VO_2peak_	NA

Pérez-Moreno et al., 2007 [26]	HIV	Aerobic capacity HRQoL	Stress test cycle ergometer	QOL	↑VO_2peak_	NS

Dolan et al., 2006 [27]	HIV	Aerobic capacity	Treadmill stress test TCAM6	NA	↑VO_2peak_ ↑TCAM6	NA

Fillipaset al., 2006 [28]	HIV	Aerobic capacity HRQoL	Kasch pulse recovery test	MOS-HIV	↓HR	↑MOS-HIV

Driscoll et al., 2004 [29]	HIV	Aerobic capacity	Submaximal stress test	NA	↑ET	NA

Rojas et al., 2003 [30]	HIV/AIDS	Aerobic capacity HRQoL	Graded exercise stress test	MOS-HIV	↑VO_2max_ ↑O_2pulse_	↑MOS-HIV

Rigsby et al., 1992 [31]	HIV	Aerobic capacity	YMCA cicle test protocol	NA	↑ET ↓HR	NA

**Table 2:** Characteristics of the experimental intervention in the trials included in the review.

Study	Type exercise	Intensity/duration (wk)	Volume	Frequency (*x* per wk)	Time (min)	Length (wk)	Supervision
Mutimura et al., 2008 [24]	Aerobic exercise	45%HR_max_/3 60% HR_max_/6 75% HR_max_/15	15 min warm-up 60 min exercise 15 min cool-down	3	90	24	Yes
	Resistance exercise	NI	NI	3	90	24	Yes

Hand et al., 2008 [25]	Aerobic exercise	50–70% HR_max_	5 min warm-up 30 min exercise 5 min cool-down	2	40	6	NR
	Resistance exercise	12 RM	1 set—12 reps	2	20	6	NR

Pérez-Moreno et al., 2007 [26]	Aerobic exercise Cycle ergometer	70–80% HR_max_	10 min warm-up 20 min exercise 10 min cool-down	3	20–40	16	Yes
	Resistance exercise	12–15 RM	1-2 set 12–15 reps	3	50	16	Yes

Fillipaset al., 2006 [28]	Aerobic exercise	60% HR_max_/3 75% HR_max_/3	5 min warm-up 20 min exercise 5 min cool-down	2	30	6	Yes
	Resistive exercise	60% MR 80% MR	3 sets 10 reps	2	30	6	Yes

Dolan et al., 2006 [27]	Aerobic exercise	60% HR_max_/2 75% HR_max_/14	5 min warm-up 20–30 min exercise	3	35	16	Yes
	Resistive exercise	60–70% MR/2 80% MR/12	3-4 sets 8–10 reps	3	85	16	Yes

Driscoll et al., 2004 [29]	Aerobic exercise Stationary bicycle	60% HR_max_/2 75% HR_max_/14	5 min warm-up 20–30 min exercise	3	35	12	Yes
	Resistance exercise	60–70% MR/4 80% MR/12	5 min cool-down 3-4 sets 8–10 reps	3	25	12	Yes

Rojas et al., 2003 [30]	Aerobic exercise	60–80% HR_max_	10 min warm-up 25 min exercise 10 min cool-down	3	50	12	NR
	Resistive exercise	60–70% MR/4 80% MR/12	2-3 sets 8 reps	3	NI	12	NR

Rigsby et al., 1992 [31]	Aerobic exercise	60–80% HR_max_	2 min warm-up 30 min exercise 3 min cool-down	3	36	12	NR
	Resistive exercise	NI	1–3 sets 6–18 reps	3	24	12	NR

NR: not reported; maximum heart rate (HR_max_); MR: maximal repetition; reps: repetitions.

**Table 3:** Effects of concurrent training on the cardiopulmonary status.

	Maximal exercise capacity			
	ΔBefore − After	*P* value	Mean difference (CI) for between-group comparison	*P* value
Mutimura et al., 2008 [24]				
Control	0.5 (0.3)	NR		
CT	4.7 (3.9)	NR	4.2 (NE)	*P* < .0001
Hand et al., 2008 [25]				
Control	0 (3.0)	NS		
CT	8.3 (3.1)	*P* < .01	NE	NE
Pérez-Morenoet al., 2007 [26]				
Control	0 (0.0)	NS	10.0 (NE)	
CT	10 (1.0)	*P* < .01		*P* < .001
Dolan et al., 2006 [27]				
Control	−2.5 (1.8)	NR		
CT	1.5 (0.8)	NR	1.0 (NE)	*P* < .001
Fillipaset al., 2006 [28]				
Control	0.6 (2.9)	NR		
CT	−19.6 (11.7)	NR	−20.2 (−25.8 to −14.6)	*P* < .001
CT	3 (0/4)	NR	3.0 (NE)	*P* < .05
Driscoll et al., 2004 [29]				
Control	0 (0/1)	NR		
CT	3 (0/4)	NR	3.0 (NE)	*P* < .05
Rojas et al., 2003 [30]				
Control	NE	NE	(NE)	(NE)
CT	2.99 (0.38)	*P* < .003		
Rigsby et al., 1992 [31]				
Control	18.18 (NR)	NR		*P* < .0001
CT	392.31 (NR)	NR	374.13 (NE)	

CT: concurrent training, NE: not estimated, NR: not reported. Data are reported as mean (SD) or as mean (95% confidence interval (CI)).
